# Association of White Blood Cell Count With Clinical Outcome Independent of Treatment With Alteplase in Acute Ischemic Stroke

**DOI:** 10.3389/fneur.2022.877367

**Published:** 2022-06-13

**Authors:** Ewgenia Barow, Fanny Quandt, Bastian Cheng, Mathias Gelderblom, Märit Jensen, Alina Königsberg, Florent Boutitie, Norbert Nighoghossian, Martin Ebinger, Matthias Endres, Jochen B. Fiebach, Vincent Thijs, Robin Lemmens, Keith W. Muir, Salvador Pedraza, Claus Z. Simonsen, Christian Gerloff, Götz Thomalla

**Affiliations:** ^1^Klinik und Poliklinik für Neurologie, Kopf- und Neurozentrum, University Medical Center Hamburg-Eppendorf, Hamburg, Germany; ^2^Hospices Civils de Lyon, Service de Biostatistique, Lyon, France; ^3^Université Lyon 1, Villeurbanne, France; ^4^Laboratoire de Biométrie et Biologie Evolutive, Equipe Biostatistique-Santé, Villeurbanne, France; ^5^Department of Stroke Medicine, Hospices Civils de Lyon, Université Claude Bernard Lyon 1, Lyon, France; ^6^Centrum für Schlaganfallforschung Berlin (CSB), Charité-Universitätsmedizin Berlin, Berlin, Germany; ^7^Medical Park Berlin Humboldtmühle, Klinik für Neurologie, Berlin, Germany; ^8^Klinik und Hochschulambulanz für Neurologie, Charité-Universitätsmedizin Berlin, Berlin, Germany; ^9^German Center for Neurodegenerative Diseases (Deutsches Zentrum für Neurodegenerative Erkrankungen), Berlin, Germany; ^10^German Center for Cardiovascular Research (Deutsches Zentrum für Herz-Kreislauf-Forschung), Berlin, Germany; ^11^Stroke Division, Florey Institute of Neuroscience and Mental Health, University of Melbourne, Heidelberg, VIC, Australia; ^12^Department of Neurology, Austin Health, Heidelberg, VIC, Australia; ^13^Department of Neurology, University Hospitals Leuven, Leuven, Belgium; ^14^Department of Neurosciences, Experimental Neurology, University of Leuven, Leuven, Belgium; ^15^Laboratory of Neurobiology, Center for Brain & Disease Research, Leuven, Belgium; ^16^Institute of Neuroscience & Psychology, University of Glasgow, Glasgow, United Kingdom; ^17^Department of Radiology, Institut de Diagnostic per la Image (IDI), Girona, Spain; ^18^Department of Neurology, Aarhus University Hospital, Aarhus, Denmark

**Keywords:** white blood cell count (WBC), ischemic stroke, treatment effect, clinical outcome, WAKE-UP, leukocyte

## Abstract

**Introduction:**

Higher white blood cell (WBC) count is associated with poor functional outcome in acute ischemic stroke (AIS). However, little is known about whether the association is modified by treatment with intravenous alteplase.

**Methods:**

WAKE-UP was a randomized controlled trial of the efficacy and safety of magnetic resonance imaging [MRI]-based thrombolysis in unknown onset stroke. WBC count was measured on admission and again at 22–36 h after randomization to treatment (follow-up). Favorable outcome was defined by a score of 0 or 1 on the modified Rankin scale (mRS) 90 days after stroke. Further outcome were stroke volume and any hemorrhagic transformation (HT) that were assessed on follow-up CT or MRI. Multiple logistic regression analysis was used to assess the association between outcome and WBC count and treatment group.

**Results:**

Of 503 randomized patients, WBC count and baseline parameters were available in 437 patients (μ = 64.7 years, 35.2% women) on admission and 355 patients (μ = 65.1 years, 34.1% women) on follow-up. Median WBC count on admission was 7.6 × 10^9^/L (interquartile range, IQR, 6.1–9.4 × 10^9^/L) and 8.2 × 10^9^/L (IQR, 6.7–9.7 × 10^9^/L) on follow-up. Higher WBC count both on admission and follow-up was associated with lower odds of favorable outcome, adjusted for age, National Institutes of Health (NIH) Stroke Scale Score, temperature, and treatment (alteplase vs. placebo, adjusted odds ratio, aOR 0.85, 95% confidence interval [CI] 0.78–0.94 and aOR 0.88, 95% CI 0.79–0.97). No interaction between WBC count and treatment group was observed (*p* = 0.11). Furthermore, WBC count on admission and follow-up was significantly associated with HT (aOR 1.14, 95% CI 1.05–1.24 and aOR 1.13, 95% CI 1.00–1.26). Finally, WBC count on follow-up was associated with larger stroke volume (aOR 2.57, 95% CI 1.08–6.07).

**Conclusion:**

Higher WBC count is associated with unfavorable outcome, an increased risk of HT, and larger stroke volume, independent of treatment with alteplase. Whether immunomodulatory manipulation of WBC count improves stroke outcome needs to be tested.

**Trial Registration:**

ClinicalTrials.gov Identifier: NCT01525290.

## Introduction

Inflammation is believed to play an important role in the pathogenesis of acute ischemic stroke (AIS) (1). A classical indicator of inflammation is the white blood cell (WBC; leukocytes) count, assessed as a routine clinical blood test. In patients with AIS, increased WBC count on admission is associated with initial stroke severity (2, 3), larger stroke lesions (4), a poor functional outcome at discharge (2), recurrent stroke, myocardial infarction, and mortality 30 days after stroke (5, 6).

In observational studies of patients treated with alteplase (recombinant human tissue plasminogen activator, rtPA), a lower WBC count on admission was associated with early neurological improvement (7) and favorable functional outcome 90 days after stroke (8, 9). Increased WBC count, measured after alteplase treatment, was associated with higher baseline National Institutes of Health Stroke Scale (NIHSS) scores (10), larger stroke volume (11), poor functional outcome, and mortality 3 months after stroke (10). Moreover, patients with increased WBC count on admission were reported to have an increased risk of symptomatic intracerebral hemorrhages (12), poor functional outcome (13), and 3-month mortality (8). Whether WBC count can also modify the treatment effect of thrombolysis has not yet been studied.

We aimed to investigate the relationship between WBC count, measured on admission and after treatment, with functional outcome, hemorrhagic transformation (HT), and stroke volume in the WAKE-UP trial (Efficacy and Safety of magnetic resonance imaging [MRI]-based Thrombolysis in Wake-Up Stroke). In addition, we aimed to study a potential interaction between WBC count and the treatment effect of intravenous alteplase.

## Materials and Methods

### Study Design

WAKE-UP was a multicenter, double-blind, placebo-controlled randomized clinical trial to study MRI-based intravenous thrombolysis in patients with AIS of unknown onset time (ClinicalTrials.gov identifier NCT01525290). The mandatory imaging criterion for randomization to treatment with alteplase or placebo was a mismatch between acute ischemic lesions on diffusion-weighted imaging (DWI) with no marked parenchymal hyperintensity on fluid-attenuated inversion recovery (FLAIR). The detailed trial protocol and the primary results were published elsewhere (14). The trial was approved for each study site by the competent authorities and the corresponding ethics committee. Patients or their legal representatives provided written informed consent according to national and local regulations, with an exception from explicit informed consent in emergency circumstances in some countries.

Following the study protocol, WBC count was measured on admission (screening prior to randomization) and 22–36 h after administration of alteplase or placebo (follow-up). Demographic data, medical history, and clinical and imaging data were assessed at baseline and follow-up. Neurological deficit was assessed upon admission by means of the NIHSS score. The outcome was assessed by the modified Rankin scale (mRS) 90 days after stroke, and a favorable outcome was defined as a mRS score of 0–1, indicating no neurological deficit or no significant disability despite minor symptoms. Further outcome measures were stroke volume and the incidence of any HT, both were measured on MRI or CT (≈6%) at 22–36 h after treatment. HT was defined as hemorrhagic infarction types 1 and 2 and parenchymal hemorrhage types 1 and 2 (15, 16).

### Statistical Analysis

White blood cell count was compared between patients with favorable and unfavorable outcome using the Wilcoxon signed-rank test. Multiple logistic regression analysis was used to assess the association between outcome and WBC count and treatment group. We fitted separate models to estimate the adjusted odds ratio (aOR) and 95% confidence interval (CI) of (i) WBC count upon admission, (ii) WBC count on follow-up, and (iii) the interaction of WBC count with treatment group on favorable outcome. We further fitted a multiple logistic regression and a linear model to estimate the influence of WBC count on HT and stroke volume, respectively. All models were adjusted for the treatment group, age, NIHSS score upon admission, and temperature. Depending on the model (WBC admission, WBC follow-up), temperature was either included upon admission or on follow-up. In a sensitivity analysis, we further adjusted all models for DWI lesion volume on admission (Supplementary Figure S3). The model on HT was adjusted for lesion volume on follow-up. Influential points, defined by the value according to the Cook's distance (>0.027), were removed from each model. All tests were carried out with a two-sided significance level of 5% without correction for multiple comparisons. For each model, we excluded patients with missing data (Supplementary Figure S1).

## Results

### Patient Characteristics

Of 503 patients randomized in WAKE-UP, 437 patients (86.9%) had available outcome, baseline data, and WBC count recordings on admission. Mean age was 64.7 (standard deviation [SD], 11.7) years, 154 (35.2%) were men. Median WBC count on admission was 7.6 × 10^9^/L (interquartile range [IQR], 6.1–9.4 10^9^/L). In total, 215 patients were assigned to receive alteplase, and 222 patients were assigned to receive a placebo. The median NIHSS score on hospital arrival was 6, (IQR 4–9). Mean body temperature was 36.5°C (SD, 0.6°C) on admission. Median lesion volume was 3.43 ml (IQR, 1.2–17.6 ml). HT occurred in 90 (22.8%) patients. In total, 355 patients (70.6%) had available outcome, baseline data, and WBC recordings on follow-up. Mean age was 65.1 (SD, 11.5) years, 121 (34.1%) were women. The median WBC on follow-up was 8.2 × 10^9^ g/l (IQR, 6.7–9.7 × 10^9^/L) with a mean body temperature of 36.8°C (SD, 0.59°C) on follow-up.

Median mRS at day 90 was 2 (IQR, 1–3), with a favorable outcome in 233 (48.8%) and unfavorable outcome (mRS 2–6) in 244 (51.2%) patients. Patients with favorable outcome were less severely affected with a median NIHSS of 5 (IQR, 3–6) when compared to patients with unfavorable outcome with a median NIHSS of 8 (IQR, 5–13, *p* < 0.001). Patients with favorable outcome presented with smaller median infarct volumes on DWI on admission with 1 ml (IQR, 1–4 ml) vs. 4 ml (IQR, 1–15 ml, *p* < 0.001) in patients with unfavorable outcome and on DWI on follow-up with 2 ml (IQR, 1–5 ml) vs. 8 ml (IQR, 2–41 ml, *p* < 0.001). Furthermore, patients with favorable outcome showed less often hemorrhages on follow-up imaging (34 [15%] patients) as compared to those with unfavorable outcome (75 [31%] patients, *p* < 0.001). All clinical characteristics for patients with favorable outcome as compared to patients with unfavorable outcome are presented in Table 1.

**Table 1:** Patient characteristics.

**Characteristics**	**mRS 0-1** ***N* = 233**	**mRS 2-6** ***N* = 244**	***P***
Age, mean (SD) [years]	64 (12)	65 (11)	0.50
Female, No. (%)	76 (33)	91 (37)	0.30
Medical history or risk factors			
Arterial hypertension, No. (%)	110 (47)	139 (57)	0.067
Diabetes mellitus, No. (%)	34 (15)	43 (18)	0.70
Hypercholesterolemia, No. (%)	71 (30)	99 (41)	0.063
Atrial fibrillation, No. (%)	21 (9)	34 (14)	0.20
WBC admission, mean (SD) [10^9^/L]	7.42 (2.44)	8.76 (3.37)	<0.001
WBC follow-up, mean (SD) [10^9^/L]	7.92 (2.25)	9.22 (2.70)	<0.001
Intravenous alteplase treatment, No. (%)	131 (56)	105 (43)	0.004
Baseline NIHSS score, median (IQR)	5.0 (3.0, 6.0)	8.0 (5.0, 13.0)	<0.001
DWI lesion volume admission, median (IQR) [ml]	1 (1, 4)	4 (1, 15)	<0.001
DWI lesion volume follow-up, median (IQR) [ml]	2 (1, 5)	9 (2, 37)	<0.001
Temperature admission, median (IQR) [C]	36.60 (36.20, 36.90)	36.40 (36.00, 36.80)	0.041
Temperature follow-up, median (IQR) [C]	36.80 (36.40, 37.10)	36.80 (36.40, 37.20)	0.5
Any hemorrhage, No. (%)	34 (15)	75 (31)	<0.001

*mRS, modified Rankin Scale; IQR, interquartile range; SD, standard deviation; NIHSS, National Institutes of Health Stroke Scale; WBC, white blood cell count; DWI, diffusion-weighted imaging*.

### Association of WBC Count With Outcome and Treatment Effect

**Figure 1:**
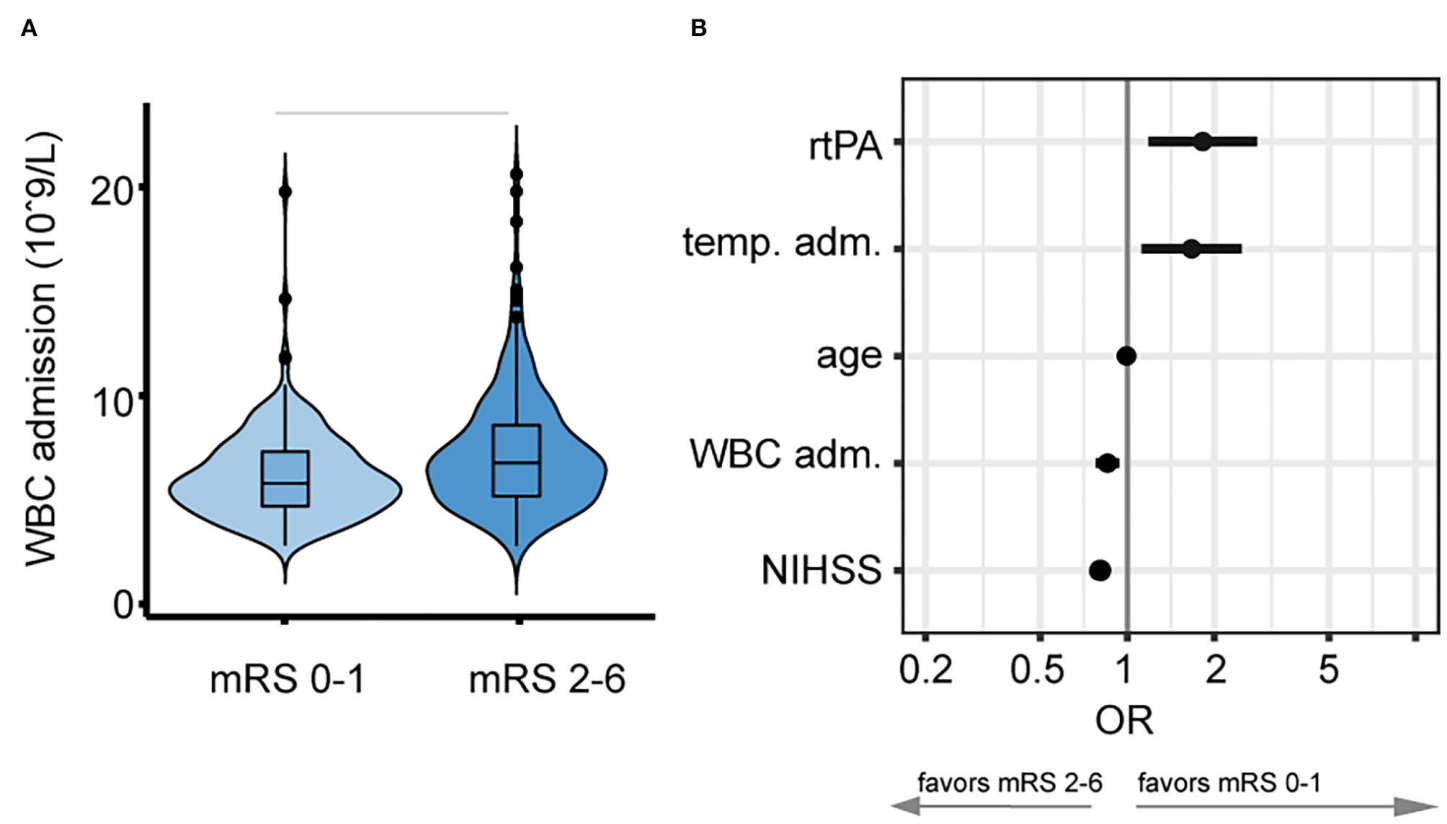
Relationship between white blood cell (WBC) count on admission and favorable outcome. **(A)** Stroke patients with a lower WBC count on admission developed better functional outcome 90 days after stroke onset. Horizontal bars indicate statistical significance for group differences (Wilcoxon test). **(B)** Lower WBC count [10^9^/L] on admission was associated with higher adjusted odds (logarithmic scale) for favorable outcome (mRS 0–1, adjusted odds ratio, and 5 and 95% CIs).

**Figure 2:**
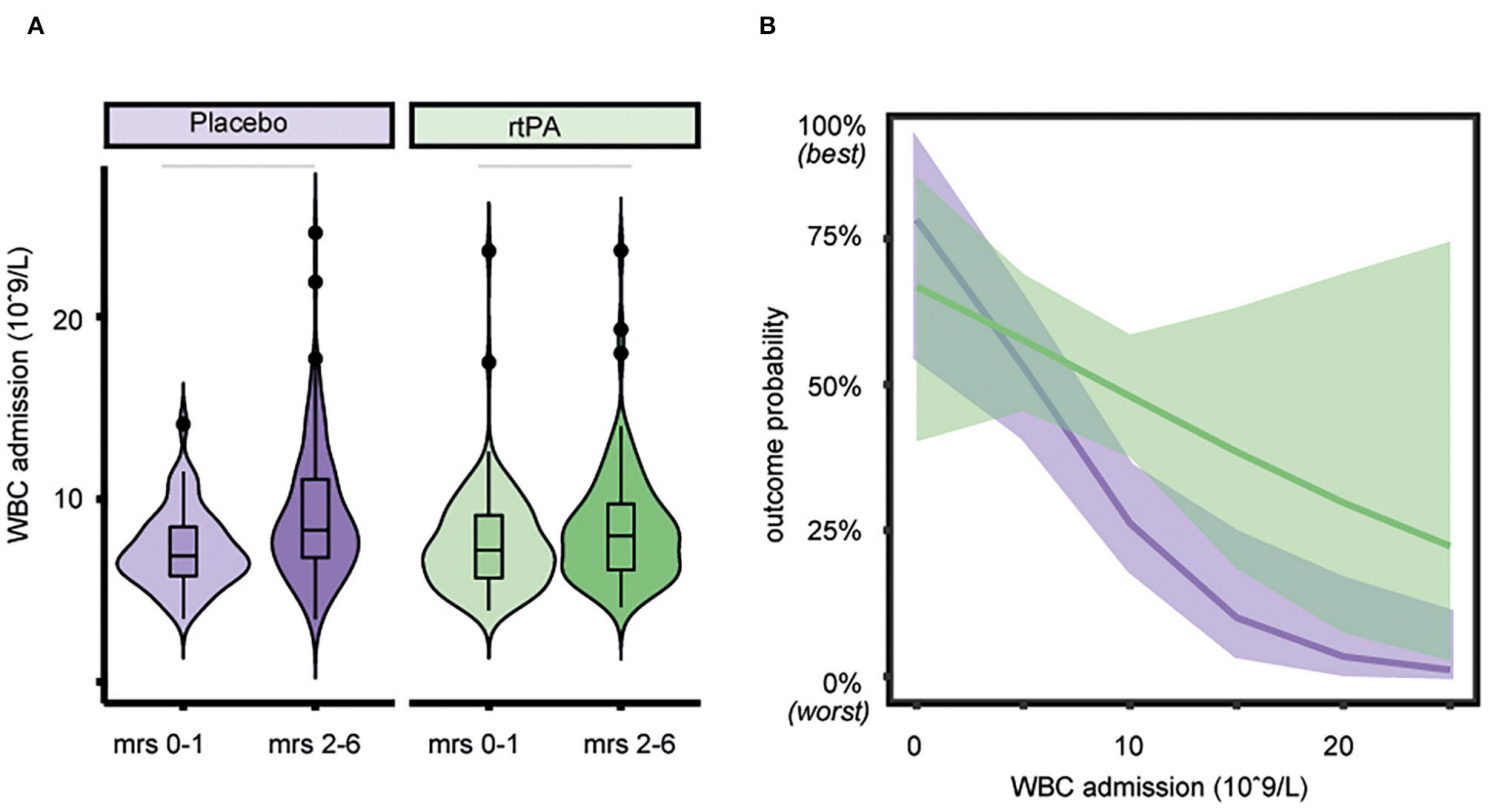
Association of white blood cell (WBC) count with outcome and treatment effect. **(A)** Lower WBC count on admission is associated with better functional outcome both in the alteplase group (*p* < 0.031), as well as in the placebo group (*p* < 0.001). Horizontal bars indicate statistical significance for group differences (Wilcoxon test). **(B)** Outcome probability of the non-significant interaction of treatment group and WBC count on admission showing a similar association of WBC count with outcome in the placebo group. Estimated outcome probability (line) and 95% CIs (shaded area), purple = placebo group, green = alteplase group.

Mean WBC count upon admission was significantly lower with 7.42 (SD, 2.44) × 10^9^/L in patients with favorable outcome (mRS 0–1) when compared to 8.76 (3.37) × 10^9^/L in patients with unfavorable functional outcome (mRS 2–6, *p* < 0.001, see Figure 1A). A higher WBC count on admission was associated with lower odds of favorable functional outcome with an aOR for favorable outcome of 0.85 (95% CI, 0.78–0.94, see Figure 1B). In patients with favorable outcome in contrast to worse outcome, a lower mean WBC count on admission was observed both in the alteplase group (7.56 [2.66] × 10^9^/L vs. 8.38 [3.10] × 10^9^/L, *p* < 0.031) and in the placebo group (7.24 [2.12] × 10^9^/L vs. 9.05 [3.54] × 10^9^/L, *p* < 0.001, see Figure 2A). In line, logistic regression analysis revealed a lower probability of favorable outcome in patients with higher WBC, adjusting for baseline parameters (aOR 0.85, 95% CI 0.77–0.93). Importantly, we did not find a significant interaction of treatment group and WBC count on admission (*p* = 0.11), indicating that the relationship of WBC count on admission and outcome was independent of alteplase treatment (see Figure 2B). Similar, a higher WBC count on follow-up was associated with lower odds of favorable outcome (aOR 0.89, 95% CI 0.80–0.98) with no interaction between WBC count at 22–36 h and treatment group (*p* = 0.67). The association of WBC count with outcome and treatment effect remained stable when further adjusting for DWI lesion volume on admission (Supplementary Figure S3).

### Association of WBC Count With Secondary Outcome

**Figure 3:**
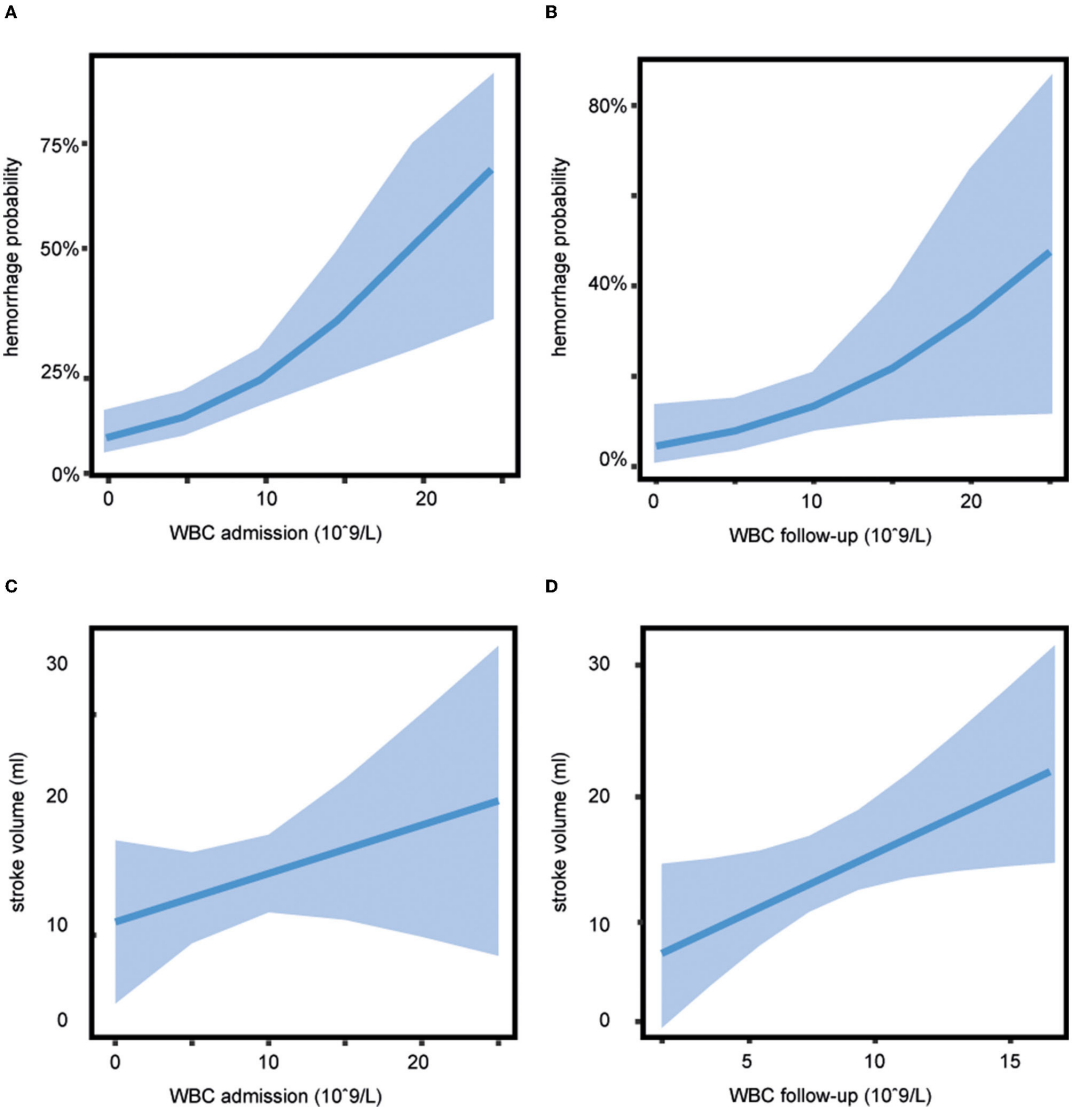
Association of white blood cell (WBC) count with secondary outcome. The probability of any hemorrhage dependent on WBC shows a greater risk of bleeding with increasing WBC count **(A)** on admission (adjusted odds ratio [aOR] 1.16, 95% CI 1.07–1.27) and **(B)** on follow-up (aOR 1.13, 95% CI 1.00–1.26), estimated hemorrhage probability (line) and 95% CIs (shaded area). **(C)** WBC count on admission was not significantly associated with stroke volume (aOR 1.55, 95% CI 0.45–4.33). **(D)** Patients with a higher WBC count on follow-up showed a larger stroke volume (aOR 2.57, 95% CI 1.08–6.07), estimated stroke volume (line), and 95% CIs (shaded area).

A higher WBC count on admission and follow-up was a significant predictor of any HT when corrected for age, temperature, and NIHSS score on admission, stroke volume, and treatment (aOR 1.16, 95% CI 1.07–1.27 for WBC count on admission, see Figure 3A, and aOR 1.13, 95% CI 1.00–1.26 for WBC count on follow-up, see Figure 3B). The association between WBC count and any HT was independent of the treatment group (*p* = 0.67 for WBC count on admission and *p* = 0.07 for WBC count on follow-up). WBC count on admission was not significantly associated with stroke volume (aOR 1.55, 95% CI 0.45–4.33, Figure 3C) whereas patients with a higher WBC count on follow-up showed a larger stroke volume (aOR 2.57, 95% CI 1.08–6.07, Figure 3D), independent of the treatment with alteplase or placebo (*p* = 0.4).

## Discussion

In this *post-hoc* analysis of the WAKE-UP trial, we studied the impact of WBC count on outcome in patients with AIS who received alteplase or placebo. Lower WBC count upon admission and 22–36 h after randomization to treatment group was associated with better functional outcome, independent of treatment. Moreover, with higher WBC count, the risk of HT was increased in both groups. Patients with a higher WBC count 22–36 h after randomization showed a larger stroke volume, independent of whether they were treated with alteplase or placebo.

Our results are in line with previous studies that observed an association between increased WBC count at the time of stroke onset with poor outcome (10, 17). Compared to previous investigations, our study is based upon a large population of acute stroke patients who were randomized in a clinical trial, which allowed a comparison of WBC count in different treatment groups. Moreover, serial measurements of WBC count were performed and thus allowed to differentiate between WBC count on admission and follow-up.

Increased WBC count may be an indicator of an activated immune system. In AIS patients, an increment of inflammatory markers may have various causes. Firstly, it can be induced by the stroke lesion itself. In the healthy brain, there are very few peripheral WBCs, but a large number of WBCs infiltrate when an ischemic stroke occurs (18). Ischemic brain lesions generate inflammatory mediators and damage-associated molecular patterns, which in turn activate the mobilization and migration of immune cells (19). The infiltration of different types of WBCs can cause deterioration through various mechanisms. Neutrophils, which are the majority of WBCs, are regarded as forerunning mediators in the ischemic brain (20, 21). The migration and accumulation of neutrophils in brain parenchyma might contribute to the disruption of the blood-brain barrier (BBB) and correlate with an increased tissue damage and poor neurological outcome (22–25). Other important immune cells are monocytes, which can favor blood coagulation by expressing thromboplastin (26). Macrophages and lymphocytes can harm the nervous system by expressing a variety of cytokines (27). Secondly, a systemic inflammatory activation in patients with AIS can be caused by secondary complications, such as post-stroke infections, which can complicate the evolution of brain tissue injury and repair and thus contribute to poor outcome (28, 29). In addition, finally, chronic comorbid diseases, such as atherosclerosis, ischemic heart disease, diabetes, hypertension, or smoking, all common in stroke patients can contribute to an increment in inflammatory markers (30–34).

The association between WBC count and functional outcome was initially described in acute stroke patients without treatment with alteplase (3). When intravenous thrombolysis with alteplase was approved and commonly practiced, a similar association was revealed in several retrospective (9, 10, 12) and prospective studies (8, 35, 36), both for WBC before treatment with alteplase and after administration of alteplase. Furthermore, the association between WBC count on admission and functional outcome was studied in one *post-hoc* analysis of the Enhanced Control of Hypertension and Thrombolysis Stroke Study (ENCHANTED) trial (13). In the ENCHANTED trial, 423 patients with increased WBC count and hyperglycemia on admission were compared to 1,831 patients with normal WBC count and normal glucose, showing the coexistence of hyperglycemia and increased WBC count was independently associated with poor functional outcome. Compared to our analysis, the conditions in that study varied significantly: both groups contained only patients who presented with a systolic blood pressure (BP; ≥150 mm Hg and who were treated with alteplase (low-dose [0.6 mg/kg] rtPA vs. standard-dose [0.9 mg/kg] alteplase). There was no control group containing patients without treatment of alteplase or with a systolic BP < 150 mm Hg on admission.

Our analysis extends these important findings by representing a more widely depicted cohort of acute stroke patients and more importantly by comparing the association between WBC count and functional outcome in a control group of acute stroke patients without treatment with alteplase with patients receiving treatment. Hence, we extend prior findings by demonstrating that the association between WBC count and functional outcome was independent of treatment.

Another finding in our analysis was that a higher WBC count on admission and follow-up was associated with an increased risk of any HT, independent of treatment. An intracranial hemorrhage is the most feared complication in the management of acute stroke patients who are eligible for thrombolysis because it can dramatically impact the prognosis (37). The results of previous studies considering the relationship between WBC count and intracranial hemorrhages, mostly assessing symptomatic intracranial hemorrhages, but also HT, are contradicting. Similar to our results, various prospective and retrospective studies have revealed that a higher WBC count was independently associated with the development of intracranial hemorrhages and thus may be partly responsible for the association of higher WBC count with poor outcome in acute stroke patients treated with alteplase (12, 35, 38). Other studies observed no relationship between WBC count and intracranial hemorrhages (8, 9), or only in patients with increased WBC count and hyperglycemia (13). Our analysis revealed an association between increased WBC count and any HT, independent of treatment with alteplase. Explanations for this association largely result from animal studies investigating immunological mechanisms. They suggest an increase of proinflammatory blood cells in acute stroke, which can either impair the BBB directly (22–24) or the function of alteplase and hence indirectly aggravate the damage of the BBB (39), which may lead to an increased risk of intracranial hemorrhages. Another study in patients with AIS with large vessel occlusion, who underwent thrombectomy, suggests that the impact of neutrophils on hemorrhagic complications and poor functional outcome depends on the extent of collaterals and reperfusion, with a more substantial impact on patients with good collaterals achieving successful reperfusion (40).

Finally, in line with previous studies, we describe an association of higher WBC count on follow-up with larger stroke volume, independent of the treatment (4, 13, 37). Based on the current state of research, a cause-effect relationship between WBC count and stroke lesion size cannot be determined, as many mechanisms may contribute to this association. Experimental observations suggest that necrosis of brain tissue, caused by acute ischemia, may induce an inflammatory activation mostly by migration and accumulation of WBCs (20). The activation of various immunological mechanisms may consecutively lead to an increase of necrotic tissue of the ischemic penumbra and thus be predictive of functional outcome (11). Neutrophils are regarded as the leading immune cells of this inflammatory response and correlate with larger infarct volume (4). After brain ischemia, a peak of neutrophil count in the peripheral blood is described at 12 h, preceding the peak in the brain lesion, which occurs within 1–2 days (41, 42). This temporal distribution of accumulating neutrophils might explain the correlation between WBC count on follow-up with stroke volume, but not with the WBC count on admission. As increased WBC count is widely known to be associated with the burden of atherosclerosis, posing a risk factor for cardiovascular events and stroke, other investigations suggest that an increment of WBC count possibly precedes stroke onset and therefore affects the stroke lesion size (31, 33, 43, 44).

Our results suggest that a higher WBC count has an important impact on functional outcome, HT, and stroke volume in acute stroke patients, independent of whether they were treated with alteplase or not. Modulation of the immune system may here be a therapeutic key target. Already existing experimental data from rodents show that pharmacological mast cell stabilization with cromoglycate can lead to a significant reduction in alteplase-mediated hemorrhagic formations and better functional outcome when compared to controls (39). A pilot trial has demonstrated that the immune modulator fingolimod plus alteplase attenuated reperfusion injury and improved clinical outcome in patients with AIS (45). Another trial studied the effect of natalizumab in patients with AIS and revealed neither an improvement in functional outcome nor a reduction of infarct growth (46, 47). However, there is no sufficient evidence to guide therapeutic interventions so far (48). Further research will be required on immunomodulation therapy targeting inflammatory pathways to prove that modulating immune response as a potential treatment for the management of patients with AIS will improve the function of alteplase and attenuate its side effects.

There are some limitations to our study. The study protocol implied routine medical care. No differentiation of leukocytes was available to determine the percentages of each type of WBCs. Therefore, our results cannot be contributed to specific WBCs but only to the WBC count in general. The assessment of the C-reactive protein (CRP) was not a part of the WAKE-UP protocol. We thus cannot rule out concomitant subclinical infection at the time of admission, which might contribute to increased WBC count in individual patients. Most patients, however, showed a WBC count within the normal range. Drugs, which might have been administered after randomization and treatment, such as antipyretics or antibiotics, were not recorded. In addition, this special patient cohort of the WAKE-UP trial differs from patient cohorts of clinical practice, e.g., patients did not exceed 80 years due to randomized-controlled trial inclusion criteria. Hence, in this study, age was not a significant predictor of clinical outcome (see Supplementary Figure S2), limiting the generalizability to real-world data. Finally, as this is a *post-hoc* analysis, all findings have to be considered hypothesis generating. Moreover, it must be acknowledged that no causality can be assumed from the detected associations. As a cause-effect relationship cannot be determined, our results do not allow a firm conclusion on management of WBC count in acute stroke patients. WAKE-UP randomized patients with unknown time of stroke onset, who, according to the DWI-FLAIR mismatch, were highly likely within 4.5 h of symptom onset. Thus, there is no reason to assume that these patients differ from patients within 4.5 h of known symptom onset with regards to the effects of intravenous alteplase.

In summary, a higher WBC count was associated with unfavorable functional outcome, increased risk of HT, and larger stroke volume in acute stroke patients, independent of treatment with alteplase.

## Data Availability Statement

Individual patients' data, after de-identification, will be accessible for researchers upon reasonable request to the corresponding author.

## Ethics Statement

The trial was approved for each study site by the competent authorities and the corresponding Ethics Committee. The Ethics Committee of the Hamburg chamber of physicians, Hamburg, Germany was the primary ethics committee that approved the trial (PVN3857). Patients or their legal representatives provided written informed consent according to National and Local Regulations, with an exception from explicit informed consent in emergency circumstances in some countries.

## Author Contributions

EB and FQ conceived and designed the study, conducted the statistical analysis, analyzed and interpreted the data, and wrote the first draft of the manuscript. BC, MG, MJ, AK, FB, MEb, MEn, JF, VT, RL, KM, NN, SP, CS, CG, and GT acquired data and critically revised the manuscript. All authors contributed to the article and approved the submitted version.

## Funding

WAKE-UP received funding from the European Union Seventh Framework Program [FP7/2007-2013] under Grant Agreement No. 278276 (WAKE-UP). This work was funded by the Deutsche Forschungsgemeinschaft (DFG, German Research Foundation), FOR 2879, TH 1106/8-1 (Grant Number 40535880).

## Conflict of Interest

EB, BC, AK, FB, NN, MEb, MEn, JF, VT, RL, KM, SP, CS, CG, and GT report grants from European Union 7th Framework Program during the conduct of the study. MEn reports grants from Bayer and fees paid to the Charité from AstraZeneca, Bayer, Boehringer Ingelheim, BMS, Daiichi Sankyo, Amgen, GSK, Sanofi, Covidien, Novartis, Pfizer and funding from DFG under Germany's Excellence Strategy – EXC-2049 – 390688087, BMBF, DZNE, DZHK, EU, Corona Foundation, and Fondation Leducq, all outside the submitted work. JF reports personal fees from Abbvie, AC Immune, Artemida, Bioclinica/Clairo, Biogen, BMS, Brainomic, Daiichi-Sankyo, Eisai, F.Hoffmann-La Roche AG, Eli Lilly, Guerbet, Ionis Pharmaceuticals, IQVIA, Janssen, Julius Clinical, Jung Diagnostics, Lysogene, Premier Research and Tau Rx, all outside the submitted work. CS reports grants from Novo Nordisk Foundation and Health Research Foundation of Central Denmark Region outside the submitted work. The remaining authors declare that the research was conducted in the absence of any commercial or financial relationships that could be construed as a potential conflict of interest.

## Publisher's Note

All claims expressed in this article are solely those of the authors and do not necessarily represent those of their affiliated organizations, or those of the publisher, the editors and the reviewers. Any product that may be evaluated in this article, or claim that may be made by its manufacturer, is not guaranteed or endorsed by the publisher.
