# Supplementary material for: Association of White Blood Cell Count With Clinical Outcome Independent of Treatment With Alteplase in Acute Ischemic Stroke
Source: Front Neurol. 2022 Jun 13;13:877367. doi: 10.3389/fneur.2022.877367 (PMC9235538; doi:10.3389/fneur.2022.877367)
Supplement: Supplementary file 1 [file Data_Sheet_1.DOCX]

Supplementary Material


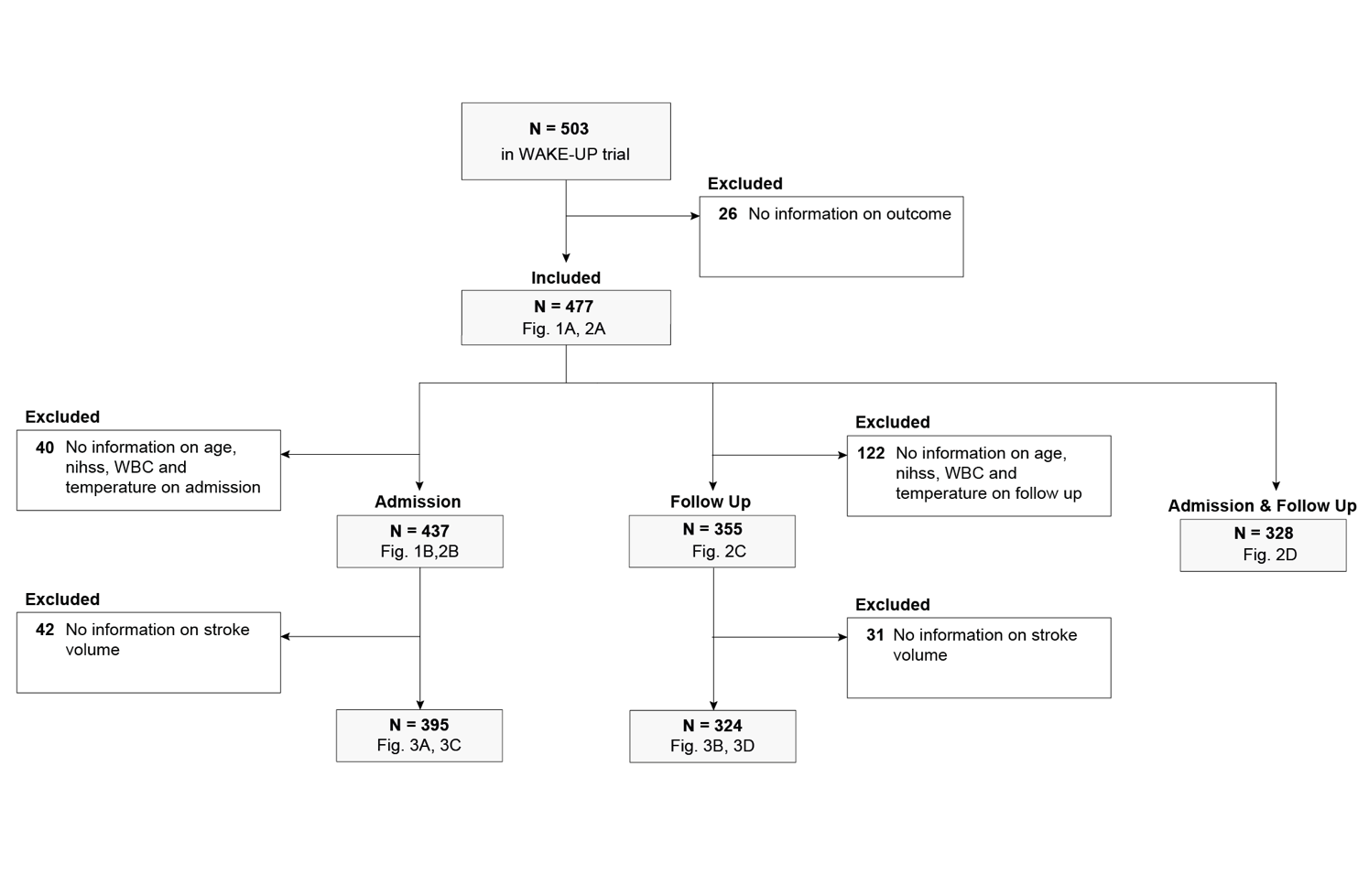


**Supplementary Figure 1.** **Patient number overview**

Flowchart of patients from WAKE-UP trial included into analyses. Different patient numbers resulted from exclusion of patients with missing data for the respective analysis.

**Supplementary Figure 2.** **Data distribution of age**

Data distribution for **(A)** age and favorable (mRS 0-1) and poor outcome (mRS 2-6), **(B)** age for each mRS and **(C)** temperature upon admission and favorable and poor outcome. Age does not significantly differ between favorable and poor outcome (*p*=0.47). In patients with favorable outcome temperature upon admission is higher compared to patients with poor outcome (*p*=0.041).


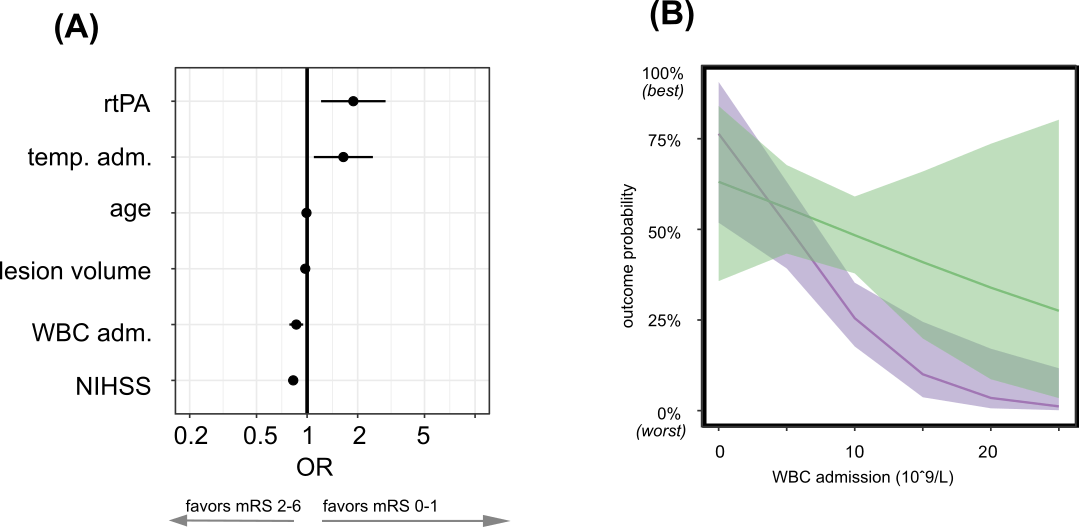


**Supplementary Figure 3. Association of WBC count with outcome and treatment effect independent of lesion volume on admission**

**(A)** Lower WBC count [10^9^/L] on admission was associated with higher adjusted odds (0.86 (95% CI, 0.78-0.95) for favorable outcome (mRS 0-1, adjusted odds ratio and 5 % and 95 % confidence intervals, logarithmic scale), also when correcting for DWI lesion volume on admission. **(B)** Outcome probability of the non-significant interaction of treatment group and WBC count (*p*=0.10) on admission, showing a similar association of WBC count with outcome in the placebo group, adjusted to initial DWI lesion volume (aOR 0.80, 95% CI 0.69-0.91). Estimated outcome probability (line) and 95 % confidence intervals (shaded area), purple = placebo group, green = alteplase group.
